# Editorial Notes

**Published:** 1938

**Authors:** 


### Editorial Notes

**The Library Association and Medical Libraries.**

The Library Association held its Annual Week-end Conference of its University and Research Section at Wills Hall from the 16th to 19th September. Among the subjects discussed was the relationship between the libraries of medical societies and Universities. In Manchester and Bristol the medical societies and the hospitals have combined with the University in forming a single medical library. The *Lancet*, in commenting on this, pays our society a generous compliment. "The Bristol Medico-Chirurgical Society," it says, "which has a fine tradition of members eminent in literature as well as in medicine, has consented to hand over its books to the University Library. . . . It is hoped, however, that medicine will soon have a separate block in the University in which the library will again become an independent unit." This hope does not imply a separate administration.

Our medical library is a sufficiently independent unit for all practical purposes. It is true that, like all other sections of the Library, it could advantageously spend more money than is at its disposal. There are great advantages in coming under the general administration of the University Librarian and his competent staff. As a separate library the staffing could never hope to be so complete.

At the moment there is no honorary medical librarian, but the office may be said to be held in commission by the three professors of medicine, surgery and obstetrics who act in an advisory capacity to the University Librarian.

Mr. W. L. Cooper is no less energetic and interested in the maintenance of an adequate medical library than in the maintenance of the other sections.

This *Journal* owes a deep debt of gratitude to the University Librarian and his whole staff. The *Lancet* concludes its notice of the Conference with the words: "In all medical libraries the staff is called upon to supply references and other material to a greater extent than in the libraries of any other faculty." This is true also of the Bristol Medical Library, and it is only just that we should acknowledge the constant help we receive from Mr. Cooper and in particular from the Senior Assistant, Mr. Roberts, who corrects all bibliographies and references that are appended to papers in this *Journal.* Moreover, he verifies every reference, which is occasionally more than the authors have done!

**The International Physicians' Luncheon Club of New York**

A MOST cordial invitation is extended to physicians visiting New York to be honoured guests at an excellent international luncheon, at the same time offering the services of the Members of the Club for any information they may desire.

While guests are not requested to make speeches, any useful information they wish to give informally will be greatly appreciated as fostering medical progress and international goodwill among physicians from all over the world.

Luncheon is served at the International Medical Center, 135 East 55th Street, New York, every Tuesday punctually at 1 o'clock and is over about 2 o'clock.

Physicians are kindly requested to inform the Club of their presence not later than 9 a.m. Tuesday by telephoning Wickersham 2–7900, or writing International Physicians' Luncheon Club, 135 East 55th Street, New York.

